# Vitamin D, vitamin A, the primary melanoma transcriptome and survival

**DOI:** 10.1111/bjd.14919

**Published:** 2016-09-26

**Authors:** S.J. O'Shea, J.R. Davies, J.A. Newton‐Bishop

**Affiliations:** ^1^ Section of Epidemiology and BiostatisticsLeeds Institute of Cancer and PathologyUniversity of LeedsCancer Genetics BuildingSt. James's University HospitalBeckett StreetLeedsLS9 7TFUK

## Abstract

Survival from melanoma is influenced by several, well‐established clinical and histopathological factors, e.g. age, Breslow thickness and microscopic ulceration. We (the Section of Epidemiology and Biostatistics, University of Leeds) have carried out research to better understand the biological basis for these observations. Preliminary results indicated a protective role for vitamin D in melanoma relapse and that higher vitamin D was associated with thinner primary melanomas.

Funding from the British Skin Foundation enabled JNB to establish a study of the effects of vitamin A in melanoma. The results suggested that vitamin A could reduce the protective effect of vitamin D in terms of overall survival. Therefore, we propose that vitamin D_3_ supplementation alone might be preferable to combined multivitamin preparations, where vitamin D supplementation is deemed to be appropriate.

Proving a causal link between vitamin D and melanoma‐specific survival is challenging. We have shown limited evidence of causation in a Mendelian randomization experiment (described in more detail later). Recent work in Leeds has also shown that higher vitamin D may be protective for microscopic ulceration. Taken together, vitamin D appears to be associated with less aggressive primary melanomas and may itself influence outcome. We continue to explore the role of vitamin D in melanoma survival and the optimum levels that might be crucial.

## Abstract 2

**What's already known about this topic?**

- Vitamin D has been reported to have anti‐proliferative effects in several cancers.
- Higher vitamin D is associated with thinner primary melanomas and a reduced risk of relapse.
- Vitamin A has been shown to antagonise vitamin D in animal models.

**What does this study add?**

- Vitamin A levels may reduce the protective effect of vitamin D on overall survival in patients diagnosed with melanoma.
- We propose supplementation of vitamin D_3_ alone in preference to multivitamins or cod liver oil, where appropriate.
- Higher vitamin D may be protective for microscopic ulceration and it is associated with less aggressive primary melanomas at a gene expression level.

In 2000, we (the Section of Epidemiology and Biostatistics, University of Leeds) started a research programme, which was intended to explore the factors associated with survival among melanoma patients. Several histopathological factors have been shown to be prognostic and, as such, have been utilized in the AJCC staging system:^1^ namely Breslow thickness, the presence of mitoses (for thin tumours, defining stage IB) and ulceration status. However, additional histopathological characteristics exist, which have been employed by other staging systems, including the presence of tumour‐infiltrating lymphocytes (TILs)^2^ and vascular invasion. Although sentinel node biopsy provides further prognostic information^3^ (which is modest taking into account all other known prognostic factors),^4^ it has no established value in terms of improving survival from melanoma. Apart from histopathological measures, clinical factors, e.g. tumour site, sex^5^ and age also have a role in determining outcome from melanoma, with male sex, older age at diagnosis and a truncal site (compared to limbs) being particularly hazardous. The underlying basis for ulceration, sex, age and site being associated with a poorer survival is not yet fully understood but these observations are clearly telling us something important about the role of the host in melanoma survival.

In an attempt to carry out research designed to increase our understanding of these tantalizing observations, in 2000 we started to build very large data and sample sets from melanoma patients. The largest of these is the Leeds Melanoma Cohort, consisting of 2184 population‐ascertained melanoma patients for which the median follow up is now 7 years.

The second dataset was a smaller case‐control study designed to identify hypotheses: comparing cases (melanoma patients at late relapse, a median of 8 years after diagnosis) with controls (melanoma patients who had survived at least 5 years without relapse). The rationale was that environmental factors could play a role in recurrence and that a comparison between late relapsers (people whose melanoma cells had by definition been relatively quiescent for many years) with non‐relapsers, might help to identify some of these elements. In 2005, an analysis of the exposure data (which had been collected by questionnaire in the case‐control study) revealed that patients who had not relapsed were more likely to have been taking vitamin D supplements than those who had progressive disease. Sixty‐two (42%) of 149 non‐relapsers and 28 (31%) of 91 relapsers reported regular intake of supplemental vitamin D 1 year before interview (OR 0·6; 95% CI, 0·4 to 1·1; *P* 0.09).^6^

Although this was a small, retrospective study, it was nonetheless an interesting observation and formed the basis of the hypothesis that vitamin D might have a role in melanoma survival. This was consistent with previous reports indicating that vitamin D was anti‐proliferative for several types of cancer *in vitro*,^7^ including melanoma.^8^ Several hypotheses regarding the mode of action of vitamin D in cancer had been postulated, including non‐genomic mechanisms, i.e. non‐vitamin D receptor‐mediated. However, vitamin D receptor (VDR) signalling is also plausible. A recent article reported that VDR signalling led to downregulation of one of the master transcription factors for cell division, *FOXM1*, and reduced growth of pancreatic ductal adenocarcinoma.^9^

We therefore went on to look at serum 25‐OH vitamin D_2/3_ levels shortly after diagnosis in relation to survival in a much larger sample from the Leeds Melanoma Cohort. This study of 872 patients, reported in 2009,^6^ showed that higher vitamin D levels were associated with thinner tumours. Higher vitamin D levels were also independently protective for relapse from melanoma. Vitamin D levels have now been measured for the entire Leeds Melanoma Cohort (*n* = 2184, as aforementioned, the median follow‐up is now 7 years). Figure 1 shows the survival rates for participants, stratified by vitamin D levels, and illustrates the melanoma‐specific survival advantage that remains for patients with higher vitamin D levels at diagnosis.

**Figure 1:**
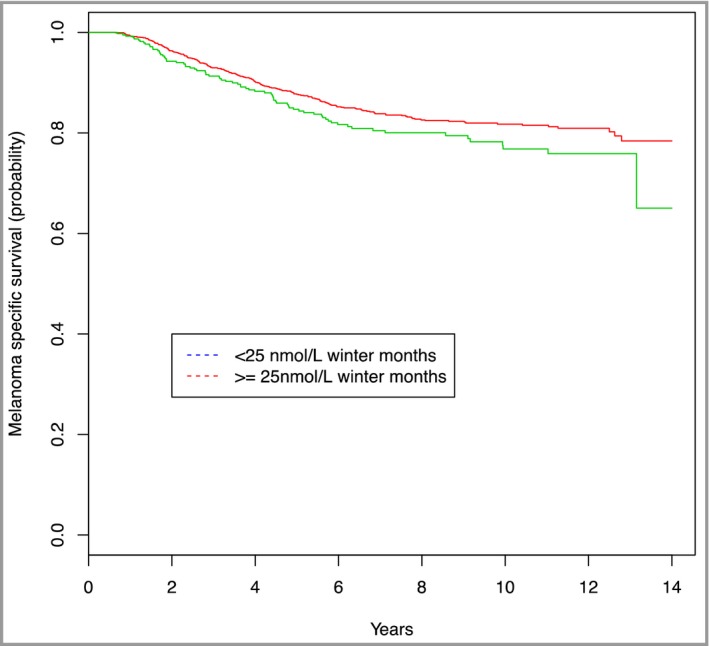
Kaplan‐Meier survival curve by serum vitamin D levels for the Leeds Melanoma Cohort Study. *Note*. This study shows differences in melanoma‐specific survival, stratified by serum vitamin D levels at diagnosis using a 25 nmol/L cut‐off point. The vitamin D levels presented here were adjusted for seasonality; serum vitamin D levels were linearly regressed on season (Jan–Mar, Apr–Jun, Jul–Sep, Oct–Dec) and batch. Levels were adjusted to blood levels as if the sample had been drawn during the winter months (Oct–Dec). Survival estimates were censored at 14 years. Logistic regression was used to adjust for sample batch and season. Participants with a vitamin D level above 25 nmol/L in these circumstances were shown to be less likely to die from their melanoma.

These two observations made in our retrospective and cohort studies suggested a role for vitamin D in melanoma survival. However, many diseases have been reported to be associated with low vitamin D levels and there has been widespread scepticism about whether or not these relationships are in fact causal. These associations could be indicative of reverse causality. Vitamin D levels are higher in leaner, fitter people so that the sceptical case is that that higher vitamin D levels may merely be acting as a marker of a healthier lifestyle. Moreover, supplementation trials have generally shown disappointing results, although a meta‐analysis of supplementation for all causes (i.e. given for any condition) did show a survival benefit.^10^ Since 2009, our group has been working to explore whether or not there is a causal relationship between vitamin D and melanoma survival and we will return to that below.

Whilst we pursued causality, JNB sought funding from the British Skin Foundation (BSF) for a study of vitamin A in melanoma patients. Vitamin D signals through the vitamin D receptor (VDR) to mediate its genomic effects, although non‐genomic effects have also been described by Deeb.^11^ The VDR however forms a heterodimer with the retinoic acid receptor, RXR, to which a derivative of vitamin A binds. We were aware of reports that vitamin A antagonises the effects of vitamin D in animal models 12 and the BSF grant sought funds to test this in the Leeds Melanoma Cohort. In this study of 795 cases,^13^ serum vitamin A levels were measured and analysed for association with Breslow thickness, overall (OS) and melanoma‐specific survival (MSS), and modification of the effect of vitamin D levels on survival. The protective effect of vitamin D on OS was reduced in patients with high vitamin A levels (≥ 2·2 μmol/l)(*HR* = 0·99, 95% CI (0·72,1·36), *P* = 0·93) compared to patients with low levels (< 2·2 μmol)(*HR =* 0·77, 95% CI (0·64, 0·93), *P* = 0·007), although the difference was not statistically significant (*P = *0·26). Our conclusion was that higher vitamin A levels may reduce the protective effect of vitamin D, although the study was likely underpowered to see a definitive effect. Sub‐optimal levels of vitamin D are common in temperate climates, and are usually managed by dietary supplementation, but vitamin A is rarely insufficient. Based on the data from the BSF funded study, we suggested that vitamin D_3_ supplementation alone might be preferable for melanoma patients than preparations containing both vitamins D and A, if supplementation seemed appropriate. In practice, this means vitamin D_3_ alone rather than cod liver oil or multivitamins.

The work we have done to explore whether there is a causal relationship between higher vitamin D levels and better melanoma survival started with an experiment known as Mendelian randomization. In this approach, inherited genetic variation, which moderates the factor under test, is sought and an association with that gene and the outcome of interest is tested. If a relationship between inheritance of that genetic variation and outcome is seen then this is good evidence that the relationship is causal (see Fig. 2a). In our experiment, we looked at the inheritance of a single nucleotide polymorphism (SNP) coding for the vitamin D binding hormone, which had been identified in genome‐wide association studies as the SNP with the strongest association with vitamin D levels 14, 15 and has been shown in our data to be so.^16^ People with this variant have lower serum levels of vitamin D. If vitamin D were important for melanoma survival then we would expect people inheriting this SNP would have a worse prognosis. We worked with colleagues from a melanoma research consortium called BioGenoMEL to carry out a meta‐analysis of data from multiple studies and showed supportive evidence for this hypothesis, although not quite conclusive.^17^ In fact only 4% of the variance in vitamin D levels in the cohort were ‘explained’ by this genetic variation,^16^ so despite recruiting several thousand patients, our study was inadequately powered to show a clear result. This is a common problem with Mendelian randomization.

**Figure 2:**
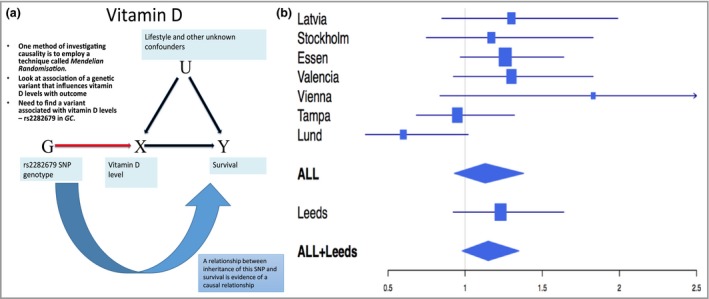
(a) illustrates how Mendelian randomization can be applied to assess whether or not a causal relationship exists between vitamin D (X) and survival (Y). Arrows highlight proposed dependency between variables. The rs2282679 SNP (G) is considered a candidate for this assessment, as it is independent of lifestyle and other confounders. (b) shows a forest plot of the hazard ratios for overall survival in the 8 cohorts (separately and combined) based on the *GC* haplotype. Cox proportional hazards models were used to generate estimates (adjusted for age, sex, site and Breslow thickness).

The most recent work was based on gene expression (transcriptomic) data from primary melanomas stored from the Leeds Melanoma Cohort participants. The primary aim of initial studies was to understand the biological basis of microscopic ulceration: a marker of poor outcome and a possible marker of intra‐tumoural inflammation. Although the significance of ulceration is strong enough to merit incorporation into the AJCC staging system, what it means biologically was unclear. It seems likely that the role of ulceration is important, supported by some evidence from an EORTC adjuvant trial which showed that only patients with ulcerated primaries appeared to benefit from treatment with pegylated interferon^18^ despite the observation that overall it is a poor prognostic factor. We had reported previously that ulcerated tumours have a more vascular stroma, rich in macrophages,^19^ suggesting the possibility that the ulcerated milieu is protumourigenic, predisposing to inflamed tumours. We therefore looked at the gene expression patterns associated with ulceration in a Leeds test set and in two validation sets from Leeds and Lund.^20^ We saw that ulceration was associated with upregulation of the pro‐inflammatory cytokines *IL‐6* and *IL‐8*. A pathway analysis was suggestive of a wound healing response, bolstering our hypothesis that inflammation is a key driver of ulcerated tumours. There is strong evidence in the literature that vitamin D levels are inversely correlated with c‐reactive protein levels (CRP),^21^ although there is controversy about the precise mechanism. Therefore, we decided to examine the relationship between vitamin D levels in the blood and ulceration. We also investigated whether or not co‐morbidities, which are known to be associated with systemic inflammation (such as diabetes mellitus, smoking and obesity), were more common in participants with ulcerated tumours. The findings confirmed this suspicion, suggesting that ulceration may be driven by systemic inflammation, but higher vitamin D levels were independently protective for ulceration.^22^

So, this first transcriptomic study, combined with the epidemiological investigation reported subsequently, suggests that vitamin D may be protective for microscopic ulceration. This is still an association and not proof that higher vitamin D levels are causally related to better survival but biologically consistent with weak previous data suggesting that vitamin D may reduce inflammatory markers.^23^, ^24^

We then argued that there would be stronger evidence of causality if serum vitamin D levels at diagnosis were associated with less aggressive biological subtypes of melanoma, especially if the genes differentially expressed were in pathways known to be downstream of the vitamin D receptor. Although research is ongoing, some preliminary evidence has indicated that higher vitamin D levels are associated with a lower probability of higher‐grade tumours^25^: that is that higher vitamin D levels appeared to be associated with less aggressive tumours at a gene expression level.

In summary then, we and now others^26^ have shown that higher vitamin D levels at diagnosis are associated with thinner primary melanomas. In addition, we and others have shown a relationship to outcome.^27^, ^28^ We have reported some evidence from a Mendelian randomization experiment of a causal effect for vitamin D and melanoma survival. This was not unequivocal: we probably needed many thousands of melanoma cases to generate conclusive results. Funding from the BSF enabled us to report evidence that higher vitamin A levels in the blood appeared to reduce the protective effect of vitamin D. We have reported evidence that higher vitamin D levels are protective for ulceration and are associated with lower grade tumours. We are building evidence for a causal relationship between low levels of vitamin D and poorer melanoma survival. We continue to work on tumour transcriptomics and vitamin D and to consider the levels of vitamin D that appear to be critical.
